# Genome-based analysis of non-ribosomal peptide synthetase and type-I polyketide synthase gene clusters in all type strains of the genus *Herbidospora*

**DOI:** 10.1186/s13104-015-1526-9

**Published:** 2015-10-09

**Authors:** Hisayuki Komaki, Natsuko Ichikawa, Akio Oguchi, Moriyuki Hamada, Tomohiko Tamura, Nobuyuki Fujita

**Affiliations:** Biological Resource Center, National Institute of Technology and Evaluation (NBRC), 2-5-8 Kazusakamatari, Kisarazu, Chiba 292-0818 Japan; NBRC, 2-49-10 Nishihara, Shibuya-ku, Tokyo, 151-0066 Japan

**Keywords:** *Herbidospora cretacea*, *Herbidospora mongoliensis*, *Herbidospora yilanensis*, *Herbidospora daliensis*, *Herbidospora sakaeratensis*, Genome sequence, Type-I polyketide synthase, Non-ribosomal peptide synthetase

## Abstract

**Background:**

The genus *Herbidospora* comprises actinomycetes belonging to the family *Streptosporangiaceae* and currently contains five recognized species. Although other genera of this family often produce bioactive secondary metabolites, *Herbidospora* strains have not yet been reported to produce secondary metabolites. In the present study, to assess their potential as secondary metabolite producers, we sequenced the whole genomes of the five type strains and searched for the presence of their non-ribosomal peptide synthetase (NRPS) and type-I polyketide synthase (PKS) gene clusters. These clusters are involved in the major secondary metabolite–synthetic pathways in actinomycetes.

**Results:**

The genome sizes of *Herbidospora cretacea* NBRC 15474^T^, *Herbidospora mongoliensis* NBRC 105882^T^, *Herbidospora yilanensis* NBRC 106371^T^, *Herbidospora daliensis* NBRC 106372^T^ and *Herbidospora sakaeratensis* NBRC 102641^T^ were 8.3, 9.0, 7.9, 8.5 and 8.6 Mb, respectively. They contained 15–18 modular NRPS and PKS gene clusters. Thirty-two NRPS and PKS pathways were identified, among which 9 pathways were conserved in all 5 strains, 8 were shared in 2–4 strains, and the remaining 15 were strain-specific. We predicted the chemical backbone structures of non-ribosomal peptides and polyketides synthesized by these gene clusters, based on module number and domain organization of NRPSs and PKSs. The relationship between 16S rRNA gene sequence-based phylogeny of the five strains and the distribution of their NRPS and PKS gene clusters were also discussed.

**Conclusions:**

The genomes of *Herbidospora* strains carry as many NRPS and PKS gene clusters, whose products are yet to be isolated, as those of *Streptomyces*. *Herbidospora* members should synthesize large and diverse metabolites, many of whose chemical structures are yet to be reported. In addition to those conserved within this genus, each strain possesses many strain-specific gene clusters, suggesting the diversity of these pathways. This diversity could be accounted for by genus-level vertical inheritance and recent acquisition of these gene clusters during evolution. This genome analysis suggested that *Herbidospora* strains are an untapped and attractive source of novel secondary metabolites.

## Background

Actinomycetes are rich sources for bioactive secondary metabolites. In particular, members of the genus *Streptomyces* have attracted attention as the most useful screening sources for new drug leads. Since the discovery of streptomycin from *Streptomyces griseus*, a large number of antibiotics have been identified from cultures of this genus [1, 2]. Consequently, the chance of finding novel secondary metabolites from *Streptomyces* members has recently dwindled. Thus, the focus of screening has moved to less exploited genera of rare actinomycetes. For example, members of the family *Streptosporangiaceae* are reported to be a promising source, and many novel compounds have been isolated from genera such as *Streptosporangium* in this family [3].

The genus *Herbidospora* was established as a new genus of the family *Streptosporangiaceae* in 1993 and currently contains five species: *Herbidospora cretacea*, *Herbidospora yilanensis*, *Herbidospora daliensis,Herbidospora sakaeratensis* and *Herbidospora mongoliensis* [4–7]. Although this genus belongs to the family *Streptosporangiaceae*, no secondary metabolites have been reported from *Herbidospora* strains in over 20 years, which motivated us to assess the potential of *Herbidospora* members as secondary metabolite producers.

Recent genome projects of actinomycetes revealed that each actinomycete genome encodes various biosynthetic pathways, and half to three quarters are associated with non-ribosomal peptide synthase (NRPS) and polyketide synthase (PKS) pathways [8]. This suggested that non-ribosomal peptide and polyketide compounds are the major secondary metabolites of actinomycetes [8]. Non-ribosomal peptides, polyketides and their hybrid compounds often show pharmaceutically useful bioactivities, many of which have been developed into various drugs, such as antibiotics, anticancer agents and immunosuppressants. Therefore, NRPS and PKS genes in actinomycete strains are often assessed to screen potential secondary metabolite producers [9, 10].

Genes for each non-ribosomal peptide and/or polyketide synthesis are generally organized into a gene cluster, in which NRPS and PKS genes play main roles to synthesize non-ribosomal peptides and polyketide chains, respectively. NRPSs and type-I PKSs are mega-synthases, containing multiple catalytic domains organized into modules, where each module carries out a cycle of chain elongation. Typically, each module contains at least three domains: a condensation (C) domain, an adenylation (A) domain and a thiolation (T) domain in NRPS modules; and a ketosynthase (KS) domain, an acyltransferase (AT) domain and an acyl carrier protein (ACP) in type-I PKS modules. Optional domains may also be present in each module to chemically modify elongating chains. The products are synthesized from simple building blocks such as acyl-CoA and amino-acid units based on an accepted theory called the assembly line rule [11]; therefore, the chemical structures of synthesized peptides and/or polyketide backbones can be predicted from domain organizations of the NRPS and/or PKS gene clusters, respectively.

In this study, we sequenced the whole genomes of all type strains of the genus *Herbidospora* because no *Herbidospora* genome sequence was registered in public databases when we began this study. We then examined the NRPS and type-I PKS gene clusters in the genome sequences and predicted the chemical backbone structures of these metabolites to assess the potential of the genus as secondary metabolite producers, and to provide information on the novelty and diversity of NRPS and PKS pathways. We also discussed how diversity was acquired during the evolution of *Herbidospora* species, based on the relationship between the distribution of these pathways and the taxonomic position of each strain.

## Methods

### Whole-genome sequencing

Genomic DNAs of *H. cretacea* NBRC 15474^T^, *H. mongoliensis* NBRC 105882^T^, *H. yilanensis* NBRC 106371^T^, *H. daliensis* NBRC 106372^T^ and *H. sakaeratensis* NBRC 102641^T^ were prepared from liquid-dried cells in ampoules provided from the NBRC culture collection, using a Qiagen EZ1 tissue kit and an EZ1 advanced instrument (Qiagen), and sequenced using paired-end sequencing with MiSeq (Illumina). The sequence redundancy for the five draft genomes ranged from 61.7 to 70.6. The sequence reads were assembled using Newbler version 2.6 software and subsequently assessed using GenoFinisher software [12].

### Analysis of NRPS and type-I PKS gene clusters

Coding sequences in the draft genome sequences were predicted using Prodigal version 2.6 [13]. NRPS and type-I PKS gene clusters were determined as previously reported [9, 10]. PKS and NRPS genes having only a single domain were excluded from the present analysis, because we considered them atypical; we focused on multi-domain genes.

### Searches for orthologous gene clusters among strains

A BLASTP search was performed using the NCBI Protein BLAST program against the non-redundant protein sequence database. We considered genes of distinct strains to be orthologous when their closest homologs in the BLASTP search were the same, and also when their domain organizations were identical or almost the same.

### Prediction of metabolites derived from NRPS and/or type-I PKS gene clusters

We used antiSMASH [14], a website for antibiotics and secondary metabolite analysis, to predict substrates for A domains and AT domains. Based on the substrates and the assembly line rule [11], we predicted the amino acid combinations of peptide chains and chemical structures of polyketide chains synthesized by NRPS and type-I PKS gene clusters, respectively.

### Phylogenetic tree based on 16S rRNA gene sequences

16S rRNA gene sequences were downloaded from ‘Sequence Information’ of the NBRC Culture Catalogue [15], and aligned using ClustalX2 [16]. A phylogenetic tree was reconstructed by the neighbor-joining method [17]. The resultant tree topologies were evaluated by bootstrap analysis [18]. The 16S rRNA gene sequence of *Acrocarpospora corrugata* NBRC 13972^T^ was used as the outgroup.

## Results and discussion

We sequenced the whole genomes of all the type strains in the genus *Herbidospora*. The genome sizes ranged from 7.9 to 9.0 Mb, showing medium size compared with those of *Streptomyces* strains (5.0–11.9 Mb) and of strains in the family *Streptosporangiaceae* (5.5–13 Mb). The five strains each possessed 15–18 gene clusters for NRPS, PKS/NRPS hybrid and type-I PKS pathways, which were similar to the numbers found in *Streptomyces* [8, 10, 19–22]. The numbers of the three types of gene clusters in each strain are listed in Table 1. Table 2 shows details of all the clusters found in each genome. Orthologous genes and gene clusters are aligned in the same row of the table. These orthologous genes showed the same domain organization; therefore, their gene clusters should synthesize the same products, as shown in the ‘Presumable product’ column of Table 2. Among the 32 gene clusters (*nrps*-*1* to -*16*, *pks/nrps*-*1* to -*4*, *pks*-*1* to -*12*) identified from the 5 strains, 9 were conserved in all strains, 8 were shared in 2–4 strains, and 15 were strain-specific. During this study, the draft genome sequence of *H. cretacea* NRRL B-16917 was published in GenBank/EMBL/DDBJ databases (accession no., JODQ00000000.1). However, it is questionable whether strain NRRL B-16917 is *H. cretacea*, because its 16S rRNA gene showed higher sequence similarity to those of type strains of *H. yilanensis* (99.1 %), *H. sakaeratensis* (98.8 %), *H. daliensis* (98.3 %) than to the type strain of *H. cretacea* (98.0 %), and its phylogenetic position was not close to the type strain of *H. cretacea* in the phylogenetic tree based on 16S rRNA gene sequences (data not shown). The scientific name of strain NRRL B-16917 is unclear; therefore, we did not analyze its NRPS and PKS gene clusters and focused on those of the five type strains in the present study.

**Table 1:** Genome sequencing and numbers of modular non-ribosomal peptide synthetase (NRPS) and type-I polyketide synthase (PKS) gene clusters in *Herbidospora* strains

Strain	Reads (Mb)	No. of scaffolds	Genome size (bp)	G + C content (%)	Accession no.	Number of gene clusters			
						NRPS	PKS/NRPS hybrid	PKS	Total
*H. cretacea* NBRC 15474^T^	582.1	36	82,82,092	70.7	BBXG01000000	9	1	5	15
*H. mongoliensis* NBRC 105882^T^	556.2	47	90,71,776	69.4	BBXD01000000	9	2	6	17
*H. yilanensis* NBRC 106371^T^	546.7	67	78,57,004	70.7	BBXE01000000	9	2	4	15
*H. daliensis* NBRC 106372^T^	586.2	18	85,23,669	70.8	BBXF01000000	10	1	4	15
*H. sakaeratensis* NBRC 102641^T^	590.3	35	83,49,170	70.9	BBXC01000000	12	2	4	18

**Table 2:** Open reading frames encoding multidomain NRPSs and PKSs in modular NRPS and PKS gene clusters of *Herbidospora* strains

Gene cluster	Scaffold-orf no. (size, % identity/similarity to closest homolog)					Domain organization	Presumed product	Closest homolog	
	*H. cretacea* NBRC 15474^T^	*H. mongoliensis* NBRC 105882^T^	*H. yilanensis* NBRC 106371^T^	*H. daliensis* NBRC 106372^T^	*H. sakaeratensis* NBRC 102641^T^			Accession no.	Origin
*nrps*-*1*	s01-orf204 (6699aa, 88/92)	s02-orf3 (6749aa, 85/90)	s34-orf56 (6711aa, 92/94)	s03-orf732 (6697aa, 89/93)	s01-orf733 (6657aa, 91/94)	A/MT/T-C/A_ser_/T-C/A_orn_/MT/T-C/A/T-C/A_ser_/T-C/A/T/E	x-Ser-mOrn-x-Ser-x (albachelin-like siderophore)	WP_034384656	*Herbidospora cretacea* NRRL B-16917
*nrps*-*2*	s17-orf53* (1026aa, 91/93)	s03-orf338* (988aa, 89/92)	s13-orf130* (1010aa, 93/94)	s05-orf276* (1051aa, 91/94)	s15-orf51* (974aa, 91/94)	A/T-C	x-Gly-Lys-x	WP_030456013	*Herbidospora cretacea* NRRL B-16917
	s17-orf54 (973aa, 88/92)	s03-orf339 (990aa, 86/91)	s13-orf129 (1015aa, 90/92)	s05-orf275 (974aa, 90/93)	s15-orf52 (975aa, 91/94)	C/A_gly_/T		WP_030456012	*Herbidospora cretacea* NRRL B-16917
	s17-orf55 (979aa, 88/90)	s03-orf340 (991aa, 85/89)	s13-orf128 (972aa, 90/92)	s05-orf274 (961aa, 91/93)	s15-orf53 (961aa, 91/93)	C/A_(lys)_/T		WP_034385955	*Herbidospora cretacea* NRRL B-16917
	s17-orf58 (1351aa, 88/92)	s03-orf343 (1341aa, 89/93)	s13-orf125 (1348aa, 94/96)	s05-orf271 (1343aa, 92/95)	s15-orf56 (1350aa, 92/95)	C/A/T-TE		WP_030456008	*Herbidospora cretacea* NRRL B-16917
*nrps*-*3*	s08-orf344 (2105aa, 86/90)	s17-orf93 (2104aa, 85/90)	s12-orf143 (2199aa, 90/91)	s01-orf374 (2125aa, 89/92)	s17-orf87 (2150aa, 90/92)	A_gly_/T-C/T-C/A_gly_/T	Gly-?-Gly-Asp	WP_034384991	*Herbidospora cretacea* NRRL B-16917
	s08-orf343 (1775aa, 91/95)	s17-orf92 (1766aa, 86/92)	s12-orf144 (1766aa, 95/97)	s01-orf373 (1776aa, 92/96)	s17-orf86 (1777aa, 92/95)	C/A_asp_/T-TE		WP_030453964	*Herbidospora cretacea* NRRL B-16917
*nrps*-*4*	s01-orf4* (1934aa, 88/91)	s02-orf218* (1932aa, 88/93)	s08-orf109* (1933aa, 95/97)	s03-orf522* (1932aa, 93/95)	s01-orf545* (1932aa, 94/95)	C/A/T-C/A_lys_/T	x-Lys-Asp	WP_030453192	*Herbidospora cretacea* NRRL B-16917
	s01-orf5 (1721aa, 93/96)	s02-orf217 (1722aa, 90/94)	s08-orf108 (1721aa, 96/98)	s03-orf523 (1721aa, 94/96)	s01-orf546 (1721aa, 95/96)	C/A_asp_/T-TE		WP_030453193	*Herbidospora cretacea* NRRL B-16917
*nrps*-*5*	s03-orf142 (1742aa, 87/91)	s04-orf515 (1751aa, 86/91)	s21-orf82 (1738aa, 95/97)	s01-orf929 (1731aa, 91/93)	s02-orf479 (1729aa, 91/94)	C/A_asp_/T-TE	Gly-Asp	WP_030454483	*Herbidospora cretacea* NRRL B-16917
	s03-orf139 (1011aa, 93/95)	s04-orf518 (1011aa, 93/95)	s21-orf85 (1011aa, 96/97)	s01-orf932 (1011aa, 93/96)	s02-orf482 (1011aa, 94/96)	C/A_gly_/T		WP_034384898	*Herbidospora cretacea* NRRL B-16917
*nrps*-*6*	s06-orf42* (474aa, 87/91)	s05-orf174* (470aa, 86/90)	s06-orf123* (471aa, 92/94)	s14-orf189* (471aa, 92/93)	s12-orf45* (471aa, 92/94)	C/T	?-Asn	WP_030450820	*Herbidospora cretacea* NRRL B-16917
	s06-orf40 (1678aa, 57/68)	s05-orf172 (1716aa, 55/66)	s06-orf125 (1684aa, 57/68)	s14-orf187 (1677aa, 57/67)	s12-orf47 (1677aa, 57/67)	C/A_asn_/T-TE		WP_031172905	*Streptosporangium roseum* NRRL B-2638
*nrps*-*7*	s05-orf29 (1709aa, 91/94)	s02-orf251 (1777aa, 90/94)	s08-orf140 (1776aa, 96/97)	s03-orf492 (1789aa, 94/96)	s01-orf513 (1812aa, 94/95)	C/A_asn_/T-TE	(Asn)	WP_030453154	*Herbidospora cretacea* NRRL B-16917
*nrps*-*8*	–	–	s05-orf295 (1018aa, 92/94)	s03-orf778 (1021aa, 89/92)	s01-orf772 (1019aa, 89/91)	A/T-TE	x–x	WP_030453437	*Herbidospora cretacea* NRRL B-16917
			s05-orf293 (1097aa, 95/97)	s03-orf780 (1097aa, 93/96)	s01-orf774 (1097aa, 94/96)	C/A/T		WP_030453439	*Herbidospora cretacea* NRRL B-16917
*nrps*-*9*	–	–	s02-orf346 (3681aa, 56/63)	–	s02-orf28 (3673aa, 53/63)	A_gly_/T-C/A_asn_/T-C/A/T-C/A_(lys)_/T	Gly-Asn-x-Lys-Thr-x	WP_026126874	*Nocardiopsis xinjiangensis* YIM 90004
			s02-orf347 (2407aa, 59/70)		s02-orf27 (2385aa, 59/70)	C/A_thr_/T-C/A/T-TE		WP_040918882	*Saccharomonospora glauca* K62
*nrps*-*10*	s05-orf197* (1110aa, 58/67)	s02-orf401* (1110aa, 58/68)	–	–	–	T-C/A_ser_/T	x-Ser-?-x–x-Ser	WP_037800301	*Streptomyces* sp. Mg1
	s05-orf195* (1679aa, 37/48)	s02-orf399* (1679aa, 37/48)				C/T-C/A/T		AGC43421	*Myxococcus stipitatus* DSM 14675
	s05-orf184 (540aa, 39/52)	s02-orf388 (527aa, 38/50)				A/T		WP_017558240	*Nocardiopsis baichengensis* YIM 90130
	s05-orf182 (1693aa, 48/57)	s02-orf386 (1700aa, 46/57)				C/T-C/A_ser_/T-TE		ACU38342	*Actinosynnema mirum* DSM 43827
*nrps*-*11*	s08-orf166 (1237aa, 63/71)	s13-orf80 (1230aa, 65/73)	–	–	–	C/A/T-TE	x	ACU75141	*Catenulispora acidiphila* DSM 44928
***nrps*** **-** ***12***	–	–	–	s01-orf155 (3126aa, 96/97)	–	C/A_phe_/T-C/A_asp_/T-C/A_asp_/T	Phe-Asp-Asp-x-Asp-Ser-x–x-Phe-x-Val-x-Tyr-Asp-Asn-Tyr-x-Tyr-Asp–Asp-x-Asp	WP_034385663	*Herbidospora cretacea* NRRL B-16917
				s01-orf140 (11331aa, 55/65)	–	C/A/T-C/A_asp_/T-C/A_ser_/T-C/A/T-C/A/T-C/A_phe_/T-C/A/T-C/A_val_/T-C/A/T-C/A_tyr_/T-C/A_asp_/T		EIF87998	*Streptomyces tsukubensis* NRRL 18488
				s01-orf139 (8503aa, 54/64)	–	C/A_asn_/T-C/A_tyr_/T-C/A/T-C/A_tyr_/T-C/A_asp_/T-C/A_asp_/T-C/A/T-C/A_asp_/T-TE		EIF87998	*Streptomyces tsukubensis* NRRL 18488
***nrps*** **-** ***13***	–	–	–	s02-orf353 (1637aa, 51/59)	–	C/A_gly_-C/A_ala_/T	Gly-Ala	WP_026213748	*Nonomuraea coxensis* DSM 45129
***nrps*** **-** ***14***	–	–	–	–	s01-orf286 (2965aa, 39/50)	C/A/T-C/A/T-C/A_ser_/T	Val-x-x-Ser-Asn–Asn	ERK92233	*Myxococcus* sp. (contaminant ex DSM 436)
					s01-orf285 (2462aa, 56/67)	C/A_asn_/T-C/A_asn_/T-C		WP_039739900	*Saccharomonospora halophila* 8
					s01-orf277* (620aa, 52/63)	A_val_/T		WP_033666206	*Salinispora pacifica* CNS055
***nrps*** **-** ***15***	–	–	–	–	s13-orf204 (1086aa, 58/66)	A_gly_/C/T	?-Gly	ETK35217	*Microbispora* sp. ATCC PTA-5024
***nrps*** **-** ***16***	–	–	–	–	s14-orf131 (1233aa, 66/75)	C/A/T	x	ELS56639	*Streptomyces viridochromogenes* Tue57
*pks/nrps*-*1*	s04-orf226 (3534aa, 82/87)	s07-orf8 (3458aa, 82/87)	s05-orf58 (3534aa, 90/92)	s06-orf132 (3570aa, 84/88)	s01-orf1038 (3515aa, 86/89)	CoL/ACP-KS/AT/KR/ACP-C/A/T-C	?-pk-x	WP_034384725	*Herbidospora cretacea* NRRL B-16917
***pks/nrps*** **-** ***2***	–	s11-orf48 (1776aa, 52/66)	–	–	–	KS/AT/DH/KR/ACP	pk-x-x–x-Val-x-x-Ser-Thr-Asn-Asn–Asn-Thr-Asn	EPH45771	*Streptomyces aurantiacus* JA 4570
		s11-orf45 (1637aa, 50/59)				A/T-C/A/T		EPH45774	*Streptomyces aurantiacus* JA 4570
		s11-orf44 (1073aa, 71/76)				C/A/T		CAC01623	*Planobispora rosea* ATCC 53733
		s11-orf43 (2007aa, 50/60)				C/A_val_/T-C/A/T		EPH43046	*Streptomyces aurantiacus* JA 4570
		s11-orf41 (2145aa, 47/57)				C/A/T-C/A_ser_/T		EPH43048	*Streptomyces aurantiacus* JA 4570
		s11-orf40 (3193aa, 41/54)				C/A_thr_/T-C/A_asn_/T-C/A_asn_/T		WP_018350842	*Longispora albida* DSM 44784
		s11-orf39 (1064aa, 45/58)				C/A_asn_/T		WP_030327232	*Streptomyces* sp. NRRL B-3229
		s11-orf38 (2516aa, 44/55)				C/A_thr_/T-C/A_asn_/T-TE		EPH43049	*Streptomyces aurantiacus* JA 4570
***pks/nrps*** **-** ***3***	–	–	s05-orf134 (1316aa, 44/56)	–	–	KS/AT/ACP-TE	Leu-Val-Leu-Ser-pk	EWC62839	*Actinokineospora* sp. EG49
			s05-orf130 (1006aa, 42/59)			A_leu_/T/E		BAH43926	*Brevibacillus brevis* NBRC 100599
			s05-orf129 (3118aa, 46/60)			C/A_val_/T-C/A_leu_/T-C/A_ser_/T		AGC43421	*Myxococcus stipitatus* DSM 14675
***pks/nrps*** **-** ***4***	–	–	–	–	*s08*-*orf1 (*> *346aa, 55/61)*	…ACP	See Fig. 1h	WP_037075741	*Pseudonocardia spinosispora* DSM 44797
					s08-orf2 (6590aa, 72/79)	KS/AT_m_/DH/KR/ACP-KS/AT_m_/DH/KR/ACP-KS/AT_m_/DH/KR/ACP-KS/AT/DH		WP_042407435	*Streptacidiphilus carbonis* NBRC 100919
					s08-orf2_1 (379aa, 83/88)	KR/ACP		WP_042407435	*Streptacidiphilus carbonis* NBRC 100919
					s08-orf3 (4716aa, 57/67)	KS/AT/KR/ACP-KS/AT/KR/ACP-KS/AT/KR/ACP-TE		WP_037075679	*Pseudonocardia spinosispora* DSM 44797
					s08-orf8 (7045aa, 69/77)	KS/AT_m_/ACP-KS/AT/DH/ER/KR/ACP-KS/AT/DH/ER/KR/ACP-KS/AT/DH/ER/KR/ACP		WP_037075740	*Pseudonocardia spinosispora* DSM 44797
					s08-orf9 (5636aa, 51/62)	KS/AT/DH/ER/KR/ACP-KS/AT/DH/ER/KR/ACP-KS/AT/DH/KR/ACP		ADL46003	*Micromonospora aurantiaca* ATCC 27029
					s08-orf10* (997aa, 65/76)	A/T		WP_042397191	*Streptacidiphilus carbonis* NBRC 100919
*pks*-*1*	s14-orf123 (2788aa, 90/92)	s10-orf265 (2815aa, 89/92)	s18-orf5 (2796aa, 90/92)	s03-orf254 (2800aa, 89/91)	s01-orf250 (2782aa, 91/94)	KS/AT_m_/ACP-KS/AT_m_/DH/KR/ACP	See Fig. 1a	WP_034382778	*Herbidospora cretacea* NRRL B-16917
	s14-orf124 (1807aa, 93/95)	s10-orf264 (1806aa, 93/96)	s18-orf6 (1800aa, 94/96)	s03-orf255 (1794aa, 94/96)	s01-orf251 (1793aa, 94/95)	KS/AT/DH/KR/ACP		WP_030450097	*Herbidospora cretacea* NRRL B-16917
	s14-orf125 (1800aa, 91/93)	s10-orf263 (1801aa, 92/93)	s18-orf7 (1795aa, 92/94)	s03-orf256 (1795aa, 90/93)	s01-orf252 (1798aa, 92/93)	KS/AT_m_/DH/KR/ACP		WP_030450096	*Herbidospora cretacea* NRRL B-16917
	s14-orf126 (1567aa, 91/94)	s10-orf262 (1578aa, 92/94)	s18-orf8 (1573aa, 91/94)	s03-orf257 (1567aa, 91/94)	s01-orf253 (1595aa, 90/92)	KS/AT/KR/ACP		WP_030450095	*Herbidospora cretacea* NRRL B-16917
	s14-orf127 (3057aa, 92/)94	s10-orf261 (3070aa, 91/94)	s18-orf9 (3062aa, 92/94)	s03-orf258 (3031aa, 92/94)	s01-orf254 (3033aa, 92/94)	KS/AT/KR/ACP-KS/AT/KR/ACP		WP_030450094	*Herbidospora cretacea* NRRL B-16917
	s14-orf135* (1265aa, 94/95)	s10-orf253* (1264aa, 95/96)	s18-orf17* (1261aa, 94/96)	s03-orf266* (1264aa, 94/96)	s01-orf262* (1261aa, 95/96)	KS/KR/ACP		WP_034382545	*Herbidospora cretacea* NRRL B-16917
	s14-orf136* (1573aa, 95/97)	s10-orf252* (1575aa, 95/97)	s18-orf18* (1573aa, 95/97)	s03-orf267* (1573aa, 94/96)	s01-orf263* (1567aa, 96/97)	KS/AT/KR/ACP		WP_034382775	*Herbidospora cretacea* NRRL B-16917
*pks*-*2*	–	s04-orf63 (6103aa, 52/62)	*s12*-*orf1 (*> *2049aa, 54/63)*	s01-orf485 (6202aa, 50/60)	s26-orf2 (5982aa, 56/65)	(KS)/AT/ACP-KS/AT/DH/KR/ACP-KS/AT/DH/KR/ACP-KS/AT/DH/KR/ACP	See Fig. 1b	AEB44393	*Verrucosispora maris* AB-18-032
			*s47*-*orf2 (*> *3456aa, 53/62)*						
		s04-orf64 (3854aa, 58/66)	*s47*-*orf1 (*> *566aa, 71/78)*	s01-orf486 (3774aa, 57/64)	*s26*-*orf1 (*> *1579aa, 53/60)*	KS/AT_(m,e)_/DH/KR/ACP-KS/AT_(m)_/DH/ER/KR/ACP		ABW12874	*Frankia* sp. EAN1pec
			*s02*-*orf383 (*> *1384aa, 66/72)*		*s02*-*orf1 (*> *2192aa, 63/70)*				
		s04-orf65 (1070aa, 81/86)	s02-orf382 (997aa, 91/93)	s01-orf487 (1019aa, 87/90)	s02-orf2 (996aa, 89/91)	KS/AT/ACP		WP_030454081	*Herbidospora cretacea* NRRL B-16917
*pks*-*3*	s12-orf80 (1259aa, 88/92)	–	s09-orf200 (1258aa, 95/96)	s01-orf1205 (1258aa, 94/96)	–	KS/AT_?_/DH/ACP	?	WP_030454772	*Herbidospora cretacea* NRRL B-16917
*pks*-*4*	–	–	s27-orf91	s06-orf349 (1795aa, 53/64)	–	KS/AT/ACP/KR/DH	Enediyne	AAP92148	*Actinomadura verrucosospora* ATCC 39334
*pks*-*5*	*s04*-*orf1 (*> *901aa, 70/79)*	*s28*-*orf45 (*> *660aa, 64/73)*	–	–	–	KS/AT…	See Fig. 1c	WP_018514184	*Streptomyces* sp. ScaeMP-e10
	*s36*-*orf55 (*> *3542aa, 58/67)*	*s11*-*orf1 (*> *405aa, 54/66)*				…ACP-KS/AT/DH/KR/ACP-KS/AT/DH/KR/ACP		WP_032771883	*Streptomyces cyaneofuscatus* NRRL B-2570
		s11-orf2 (3315aa, 50/61)							
	s36-orf54 (2142aa, 66/75)	s11-orf3 (2345aa, 50/62)				KS/AT_(m)_/DH/KR/ACP		WP_032771870	*Streptomyces cyaneofuscatus* NRRL B-2570
***pks*** **-** ***6***	s01-orf132 (1020aa, 52/63)	–	–	–	–	KS/AT_m_/ACP	See Fig. 1d	EJJ02887	*Streptomyces auratus* AGR0001
	s01-orf133 (7608aa, 50/61)					KS/AT/KR/ACP-KS/AT/DH/ER/KR/ACP-KS/AT/KR/ACP-KS/AT_m_/KR/ACP-KS/AT/KR/ACP		WP_035304435	*Actinokineospora inagensis* DSM 44258
	s01-orf134 (4820aa, 54/64)					KS/AT/KR/ACP-KS/AT/DH/ER/KR/ACP-KS/AT/KR/ACP		EHY88974	*Saccharomonospora azurea* NA-128
	s01-orf135 (6313aa, 52/63)					KS/AT_m_/KR/ACP-KS/AT/DH/KR/ACP-KS/AT/DH/KR/ACP-KS/AT/DH/KR/ACP		ACU36619	*Actinosynnema mirum* DSM 43827
	s01-orf136 (4533aa, 55/65)					KS/AT/DH/KR/ACP-KS/AT/KR/ACP-KS/AT/KR/ACP		AAX98184	*Streptomyces aizunensis* NRRL B-11277
	s01-orf137 (4803aa, 55/65)					KS/AT_e(m)_/DH/KR/ACP-KS/AT_e(m)_/KR/ACP-KS/AT/DH/KR/ACP		WP_033261216	*Amycolatopsis vancoresmycina* NRRL B-24208
	s01-orf138 (5850aa, 54/65)					KS/AT/KR/ACP-KS/AT_m_/KR/ACP-KS/AT_m_/KR/ACP-KS/AT/KR/ACP		ABC87511	*Streptomyces* sp. NRRL 30748
	s01-orf139 (4995aa, 52/64)					KS/AT_m_/KR/ACP-KS/AT/DH/ER/KR/ACP-KS/AT/DH/KR/ACP		AHH99925	*Kutzneria albida* DSM 43870
	s01-orf140 (5337aa, 54/64)					KS/AT/KR/ACP-KS/AT/DH/ER/KR/ACP-KS/AT/DH/ER/KR/ACP		AEP40936	*Nocardiopsis* sp. FU40
	s01-orf141 (3460aa, 57/68)					KS/AT/DH/KR/ACP-KS/AT/DH/KR/ACP		WP_035796302	*Kitasatospora mediocidica* KCTC 9733
	s01-orf142 (4383aa, 54/65)					KS/AT/KR/ACP-KS/AT_m_/KR/ACP-KS/AT/KR/ACP		AAX98186	*Streptomyces aizunensis* NRRL B-11277
	s01-orf143 (1247aa, 53/64)					KS/AT/ACP-TE		KIR65900	*Micromonospora carbonacea* JXNU-1
***pks*** **-** ***7***	s20-orf48 (2065aa, 59/68)	–	–	–	–	KS/AT_e(m)_/ACP-KS/AT/DH/ACP	See Fig. 1e	WP_042439448	*Streptacidiphilus albus* NBRC 100918
	s20-orf49 (1530aa, 53/63)					KS/AT/KR/ACP		WP_042494385	*Streptomyces avermitilis* MA-4680 = NBRC 14893
	s20-orf50 (2820aa, 62/70)					KS/AT/DH/KR/ACP-KS/AT_m_/ACP		CCH32016	*Saccharothrix espanaensis* DSM 44229
	s20-orf51 (2064aa, 57/66)					KS/AT/DH/KR/ACP-TE		WP_041313683	*Saccharothrix espanaensis* DSM 44229
***pks*** **-** ***8***	–	s08-orf244 (5375aa, 59/69)	–	–	–	KS/AT/DH/ER/KR/ACP-KS/AT_e(m)_/KR/ACP-KS/AT_m_/DH/KR/ACP	See Fig. 1f	WP_033660827	*Salinispora pacifica* CNS237
		s08-orf245 (1559aa, 55/66)				KS/AT/KR/ACP		EXU62139	*Streptomyces* sp. PRh5
		s08-orf246 (3222aa, 53/65)				KS/AT_e(m)_/KR/ACP-KS/AT/DH/KR/ACP		EGX61520	*Streptomyces zinciresistens* K42
		s08-orf251 (10965aa, 51/63)				KS/AT/ACP-KS/AT_e(m)_/DH/KR/ACP-KS/AT_m_/KR/ACP-KS/AT/DH/KR/ACP-KS/AT_e(m)_/DH/KR/ACP-KS/AT_e(m)_/KR/ACP-KS/AT/KR/ACP		ADI03772	*Streptomyces bingchenggensis* BCW-1
		s08-orf252 (1565aa, 64/73)				KS/AT_m_/KR/ACP		WP_033775512	*Salinispora pacifica* DSM 45546
		s08-orf253 (3638aa, 59/69)				KS/AT/DH/ER/KR/ACP-KS/AT_m_/KR/ACP		CAJ88176	*Streptomyces ambofaciens* ATCC 23877
		s08-orf254 (7933aa, 67/76)				KS/AT/DH/KR/ACP-KS/AT_m_/DH/ER/KR/ACP-KS/AT/KR/ACP-KS/AT/KR/ACP-KS/AT/KR/ACP		CAJ88175	*Streptomyces ambofaciens* ATCC 23877
***pks*** **-** ***9***	–	s11-orf206 (7923aa, 54/66)	–	–	–	KS/AT/KR/ACP-KS/AT/KR/ACP-KS/AT/DH/KR/ACP-KS/AT/DH/KR/ACP-KS/AT/DH/KR/ACP	See Fig. 1g	BAC68129	*Streptomyces avermitilis* MA-4680 = NBRC 14893
		s11-orf207 (1582aa, 54/65)				KS/AT/KR/ACP		WP_032769932	*Streptomyces* sp. CNS654
		s11-orf208 (4703aa, 59/69)				KS/AT_m_/KR/ACP-KS/AT/KR/ACP-KS/AT/KR/ACP-TE		CAJ88175	*Streptomyces ambofaciens* ATCC 23877
***pks*** **-** ***10***	–	s26-orf59 (1260aa, 87/92)	–	–	–	KS/AT/ACP	?	WP_030454772	*Herbidospora cretacea* NRRL B-16917
***pks*** **-** ***11***	–	–	–	–	s22-orf6 (4492aa, 74/80)	KS/AT/DH/KR/ACP-KS/AT_m_/KR/ACP-KS/AT/KR/ACP	See Fig. 1i	WP_042397188	*Streptacidiphilus carbonis* NBRC 100919
					s22-orf5 (6067aa, 75/82)	KS/AT_m_/KR/ACP-KS/AT/KR/ACP-KS/AT_m_/KR/ACP-KS/AT/KR/ACP		WP_042397185	*Streptacidiphilus carbonis* NBRC 100919
					s22-orf4 (3381aa, 56/65)	KS/AT_m_/DH/KR/ACP-KS/AT_m_/KR/ACP		WP_037075673	*Pseudonocardia spinosispora* DSM 44797
					s22-orf3 (6184aa, 71/78)	KA/AT_m_/KR/ACP-KS/AT_m_/KR/ACP-KS/AT_m_/KR/ACP-KS/AT_m_/KR/ACP		WP_037075676	*Pseudonocardia spinosispora* DSM 44797
					s22-orf2 (6426aa, 76/83)	KS/AT_m_/KR/ACP-KS/AT_m_/KR/ACP-KS/AT/KR/ACP-KS/AT_m_/DH/KR/ACP		WP_042408959	*Streptacidiphilus carbonis* NBRC 100919
					*s22*-*orf1 (*> *4929aa, 77/83)*	KS/AT_m_/KR/ACP-KS/AT_m_/DH/ER/KR/ACP-KS/AT_m_/DH…		WP_042408956	*Streptacidiphilus carbonis* NBRC 100919
***pks*** **-** ***12***	–	–	–	–	s02-orf765 (1258aa, 94/95)	KS/AT_?_/DH/ACP	?	WP_030454772	*Herbidospora cretacea* NRRL B-16917

Strain-specific gene clusters are in boldface. NRPS and PKS genes not completely sequenced are shown in italics. C, condensation; A, adenylation; T, thiolation; E, epimerization; MT, methyltransferase; CoL, CoA ligase; KS, ketosynthase; AT, acyltransferase incorporating malonyl-CoA, ATm, acyltransferase incorporating methylmalonyl-CoA; ATe, acyltransferase incorporating ethylmalonyl-CoA; AT?, acyltransferase whose substrate has not been determined by antiSMASH; DH, dehydratase; ER, enoylreductase; KR, ketoreductase; ACP, acyl carrier protein; TE, thioesterase; x, unpredicted amino-acid; mOrn, methyl ornithine; ?, lack of A domain. A domain amino-acid substrate predicted by antiSMASH is shown in subscripted letters

### Gene clusters conserved in all the five strains

**Fig. 1:**
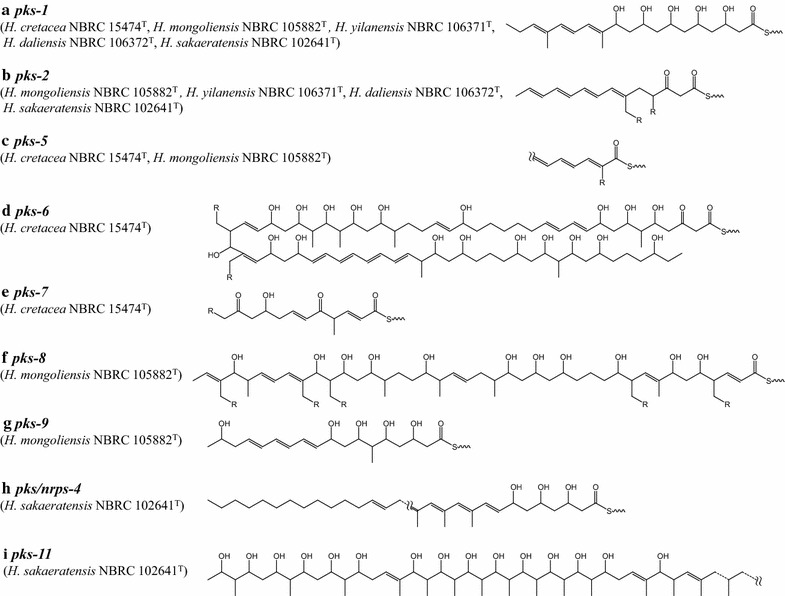
Presumed chemical structures of polyketide backbones synthesized by *Herbidospora* type-I polyketide synthase (PKS) gene clusters. R=H or methyl. The chemical structures are shown attached to the ACP of the last module by thioester bonds

Table 2 suggested that nine presumable products (*nrps*-*1* to -*7*, *pks/nrps*-*1*, *pks*-*1*) are common among all five type strains belonging to the genus *Herbidospora*. *Nrps*-*1* is assumed to be involved in the synthesis of a siderophore similar to albachelin [23], because the module numbers of albachelin NRPS and *nrps*-*1* are the same, and their domain organizations and amino-acid substrates of their A domains are quite similar (albachelin, C/A/T-C/A_Ser_/T/E-C/A_Orn_/MT/T-C/A_Ser_/T-C/A_Ser_/T-C/A/T/E; NRPS-1, A/MT/T-C/A_Ser_/T-C/A_Orn_/MT/T-C/A/T-C/A_Ser_/T-C/A/T/E. Distinct domains between them are underlined). *Nrps*-*2* to -*6* are predicted to synthesize non-ribosomal peptides comprising 4, 4, 3, 2 and 2 amino acids, respectively, based on their module numbers. *Nrps*-*7* had only a single NRPS module; therefore, we were not able to predict the chemical structure of the product as a peptide. *Pks/nrps*-*1* is a PKS/NRPS hybrid gene encoding a protein comprising three modules for the synthesis of a starter unit, a polyketide unit and an amino-acid unit, respectively. *Pks*-*1* gene clusters contained seven PKS genes, whose assembly line was composed of nine modules. According to the assembly line rule and the substrates of their AT domains, the gene clusters were assumed to synthesize the polyketide chain shown in Fig. 1a. The structure has similar characteristics to those of antifungal polyene compounds, containing multiple carbon–carbon conjugated double bonds and multiple hydroxyl groups.

### Gene clusters shared between/among two to four strains

*Nrps*-*8* gene clusters were present in three strains (*H. yilanensis* NBRC 106371^T^, *H. daliensis* NBRC 106372^T^ and *H. sakaeratensis* NBRC 102641^T^). The *nrps*-*8* gene clusters had two modules; therefore, the products were predicted to be dipeptides. *Nrps*-*9* gene clusters present in *H. yilanensis* NBRC 106371^T^ and *H. sakaeratensis* NBRC 102641^T^ possessed five modules. According to the predicted substrates of the A domains in each module, the products would be hexapeptides including glycine (Gly), asparagine acid (Asp), lysine and threonine (Thr) as the building blocks. *Nrps*-*10* and *nrps*-*11* gene clusters were present in *H. cretacea* NBRC 15474^T^ and *H. mongoliensis* NBRC 105882^T^. The *nrps*-*10* gene clusters contained six modules and two A domains were predicted to incorporate serine (Ser) as the substrates; therefore, the products were predicted to be hexapeptides including two Ser molecules. In contrast, *nrps*-*11* had only a single module, and we were not able to predict the peptide product.

*Pks*-*2* gene clusters were present in four strains, with the exception of *H. cretacea* NBRC 15474^T^, although those of *H. yilanensis* NBRC 106371^T^ and *H. sakaeratensis* NBRC 102641^T^ were not completely sequenced. The gene clusters contained seven modules, suggesting the products would be molecules derived from C_14_ polyketide chains. Substrates of AT domains in modules 5 and 6 were predicted to be methylmalonyl-CoA or ethylmalonyl-CoA, and those of all the remaining modules were malonyl-CoA. Four pairs of dehydratase (DH)-ketoreductase (KR) and one trio of DH-enoylreductase (ER)-KR were present as the optional domains in the gene clusters; therefore, four keto groups would be reduced to four conjugated double bonds and one keto group would be completely reduced, respectively. Hence, we predicted the chemical structure of the polyketide backbones shown in Fig. 1b. *Pks*-*3* genes were present in *H. cretacea* NBRC 15474^T^, *H. yilanensis* NBRC 106371^T^ and *H. daliensis* NBRC 106372^T^. They contained only a single module and showed low sequence similarities to characterized PKS genes (data not shown); therefore, we were not able to predict the metabolites. *Pks*-*4* genes were present in *H. yilanensis* NBRC 106371^T^ and *H. daliensis* NBRC 106372^T^. They were predicted to be iterative type-I PKSs for enediyne synthesis, called PksE, because they showed higher sequence similarities to PksEs than to normal modular type-I PKSs (data not shown) and included a pair of KR-DH domains, specific for PksE, after the ACP. Their products would be polyketide compounds with an enediyne core [24]. *Pks*-*5* gene clusters were present in *H. cretacea* NBRC 15474^T^ and *H. mongoliensis* NBRC 105882^T^; however, they were not completely sequenced. Hence, we were not able to predict whole chemical structure of the polyketide chain.

### Strain-specific clusters

We found 2, 4, 1, 2 and 6 strain-specific NRPS and/or PKS gene clusters in *H. cretacea* NBRC 15474^T^, *H. mongoliensis* NBRC 105882^T^, *H. yilanensis* NBRC 106371^T^, *H. daliensis* NBRC 106372^T^ and *H. sakaeratensis* NBRC 102641^T^, respectively.

*H. cretacea* NBRC 15474^T^ possessed 2 specific PKS gene clusters, named *pks*-*6* and *pks*-*7*. The *pks*-*6* gene cluster contained 35 modules encoded by 12 PKS genes. To the best of our knowledge, this is the largest type-I PKS gene cluster ever reported. We predicted the chemical structure of the polyketide backbone synthesized by *pks*-*6*, as shown in Fig. 1d, which is most likely a novel compound because no similar compounds were found in our database searches. The *pks*-*7* gene cluster contained 6 modules encoded by 4 PKS genes and we predicted the metabolites to be hexaketide compounds with 2 C–C double bonds and one hydroxyl group.

*H. mongoliensis* NBRC 105882^T^ possessed a specific PKS/NRPS gene cluster and 3 specific PKS gene clusters, named *pks/nrps*-*2*, *pks*-*8*, *pks*-*9* and *pks10*. *Pks/nrps*-*2* contained 1 PKS module and 13 NRPS modules, encoded by a PKS gene and seven NRPS genes, respectively. According to the module numbers and the A domain substrates, the product was predicted to be large polyketide-non-ribosomal peptide hybrid compound including Ser, Thr and asparagine (Asn). The *pks*-*8* gene cluster contained seven PKS genes encoding 21 modules. As shown in Fig. 1f, its products would be large polyketide compounds with 6 C–C double bonds and 11 hydroxyl groups. The *pks*-*9* contained 3 PKS genes encoding 9 modules. Its products were predicted to be nonaketide compounds with 3 conjugated double bonds and 5 hydroxyl groups (Fig. 1g). The *pks*-*10* gene encoded only a single module; therefore, we were not able to predict the chemical structure of its metabolite.

*H. yilanensis* NBRC 106371^T^ possessed a specific PKS/NRPS hybrid gene cluster named *pks/nrps*-*3*. The products were predicted to be polyketide-non-ribosomal peptide hybrid compounds whose backbone is leucine (Leu)-valine (Val)-Leu-Ser-a polyketide unit.

*H. daliensis* NBRC 106372^T^ possessed 2 specific NRPS gene clusters, named *nrps*-*12* and *nrps*-*13*. The *nrps*-*12* gene cluster encoded 22 modules, the products of which were predicted to be peptide compounds comprising 22 amino-acids, including 2 phenylalanine (Phe), 7 Asp, 1 Ser, 1 Val, 3 tyrosine (Tyr), and 1 Asn molecules. In contrast, *nrps*-*13* contained only two modules, whose A domains were predicted to incorporate Gly and Ala, respectively. Hence, the products will be dipeptides including Gly and Ala molecules.

*H. sakaeratensis* NBRC 102641^T^ possessed 3 specific NRPS gene clusters, one specific PKS/NRPS hybrid gene cluster and two specific PKS gene clusters named *nrps*-*14* to -*16, pks/nrps*-*4, pks*-*11* and *pks*-*12*. In the *nrps*-*14* gene cluster, three NRPS genes were present encoding six modules. The A domains of four modules were predicted to incorporate Val, Ser, Asn and Asn, suggesting that *nrps*-*14* would produce hexapeptides including Val, Ser and two Asn molecules. *Nrps*-*15* had only one module, but the domain organization (A-C-T) was different from that of normal NRPS (C-A-T). Because a CoA-ligase, an ACP, and an NRPS comprising only one C domain were also encoded adjacent to *nrps*-*15* gene (data not shown), this gene cluster might synthesize compounds composed of a starter molecule and a Gly molecule loaded by the unusual NRPS. *Nrps*-*16* also had only a single module; therefore, we were not able to predict its peptide products. The *pks/nrps*-*4* gene cluster encoded at least 15 PKS modules and one NRPS module, but it was not completely sequenced because the sequence of a PKS gene named s08-orf1 was partial and the adjacent genes remain unclear. Although we were not able to predict the whole chemical structure synthesized by this gene cluster, the product will include a C_28_ or longer polyketide chain. The *pks*-*11* gene cluster encoded at least twenty modules, although s22-orf1 was not completely sequenced and the adjacent genes were unclear. This product was predicted to be a large compound including a polyhydroxyl polyketide chain, as shown in Fig. 1i. The *pks*-*12* gene cluster encoded only a single module; therefore, we were not able to predict chemical structures of its products.

### Distribution and evolutionary history of NRPS and PKS gene clusters

**Fig. 2:**
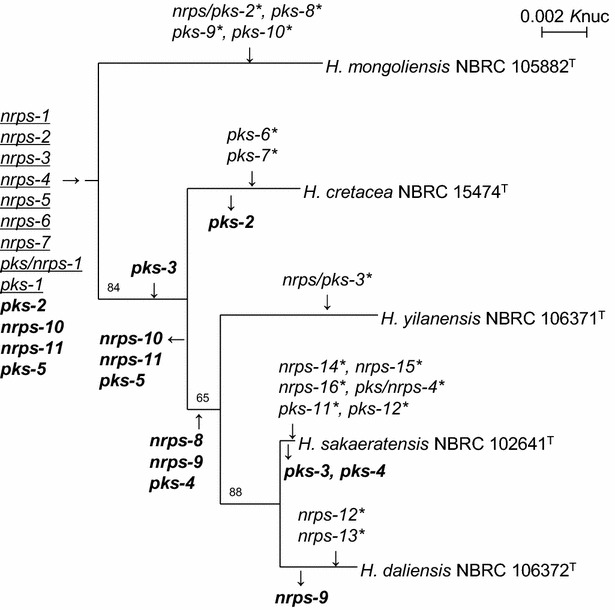
Phylogenetic tree of the type strains of the genus *Herbidospora*, based on 16S rRNA gene sequences, depicting the inferred ancestry of NRPS and PKS gene clusters. Bootstrap values (>50 %) from 1000 replicates are shown at branch nodes.* Arrows* indicate acquisitions and losses of NRPS and PKS gene clusters. Gene clusters conserved all the five strains, shared between/among two to four strains, and specific to each strain are *underlined*, *boldfaced*, and *asterisked*, respectively

**Fig. 3:**
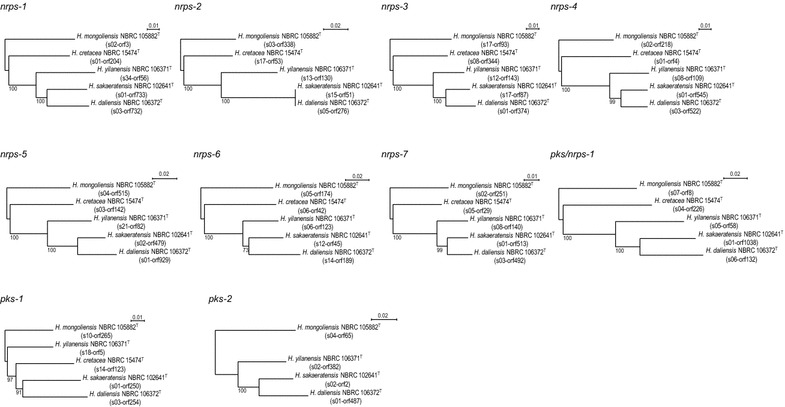
Phylogenetic tree of the type strains of the genus *Herbidospora*, based on NRPS and PKS sequences. Bootstrap values (>50 %) from 1000 replicates are shown at branch nodes. The proteins used in this analysis are shown in *parentheses* by open reading frame numbers listed in Table 2

We constructed a phylogenetic tree of the type strains of the genus *Herbidospora* based on 16S rRNA gene sequences. By mapping the inferred ancestral nodes of the individual gene clusters onto the tree, we traced the evolutionary histories of these pathways (Fig. 2). Nine gene clusters, underlined in Fig. 2, appeared to have been acquired early in the evolution of the genus *Herbidospora*, because they are conserved in all the type strains. By contrast, 15 gene clusters, indicated by asterisks in Fig. 2, would have been acquired relatively recently, appearing toward the branch terminals in the tree. Gene clusters shared between/among 2–4 strains are in boldface in Fig. 2. *Pks*-*2* gene clusters are present in 4 strains, except for *H. cretacea* NBRC 15474^T^, suggesting they were acquired early, and inherited vertically; however, they were lost just before evolution to *H. cretacea*. The *nrps*-*10*, *nrps*-*11* and *pks*-*5* clusters are present in *H. mongoliensis* NBRC 105882^T^ and *H. cretacea* NBRC 15474^T^, but not in the other 3 strains, we speculated that these 3 gene clusters were acquired early, but lost just before evolution to *H. yilanensis*, *H. sakaeratensis* and *H. daliensis*. The *pks*-*3* gene clusters are present in three strains except for *H. mongoliensis* NBRC 105882^T^ and *H. sakaeratensis* NBRC 102641^T^, suggesting that they were acquired just branching off from *H. mongoliensis* and lost just before evolution to *H. sakaeratensis*. *Nrps*-*8* gene clusters are present in *H. yilanensis* NBRC 106371^T^, *H. sakaeratensis* NBRC 102641^T^ and *H. daliensis* NBRC 106372^T^, suggesting acquisition just before evolution to these three species. Similarly, the *nrps*-*9* and *pks*-*4* gene clusters would also have been acquired at the same point; however, these clusters seemed to have been lost during evolution to *H. daliensis* and *H. sakaeratensis*, respectively. To confirm the hypothesis, we conducted phylogenetic analysis of NRPSs and PKSs in gene clusters conserved among more than 4 strains. Except for *pks*-*1*, all the phylogenetic trees showed the same topology (Fig. 3) as that based on 16S rDNA sequences (Fig. 2). This supports that *nrps*-*1* to -*7*, *pks/nrps*-*1* and *pks*-*2* were actually acquired early in the evolution and inherited vertically. In contrast, *pks*-*1* may not be inherited in the same manner as these gene clusters, because the topology of *pks*-*1* phylogenetic tree differed from those of other gene clusters and 16S rDNA sequence.

## Conclusions

We concluded the following: (1) The genomes of *Herbidospora* strains carry as many NRPS and PKS gene clusters as those of other actinomycetes such as *Streptomyces*; however, their products are yet to be isolated; (2) members of the genus *Herbidospora* can synthesize large and diverse metabolites, many of whose chemical structures are yet to be reported; (3) each strain possesses 1–6 strain-specific NRPS and/or PKS gene clusters, in addition to those conserved within this genus, suggesting diversity of these pathways; and (4) the diversity of NRPS and PKS pathways in each strain has increased by genus-level vertical inheritance and relatively recent acquisitions of these gene clusters during evolution of this genus.

To summarize, in this study, we sequenced whole genomes of all the five type strains belonging to the genus *Herbidospora* and examined their NRPS and PKS gene clusters. Each strain harbored 15–18 modular NRPS and PKS gene clusters. Through the comparison of these gene clusters, 32 NRPS and PKS pathways were identified from the 5 strains. Among them, 9 pathways were conserved in all 5 strains, 8 were shared in 2–4 strains, and the remaining 15 were strain-specific suggesting the strain diversity of these pathways. We revealed that these strains harbor a wealth of NRPS and PKS pathways, many of whose products are large and have yet to be discovered. This study also provided useful information about the inferred numbers and molecular structures of secondary metabolites, such as non-ribosomal peptides and polyketides, potentially produced by these strains, suggesting that *Herbidospora* strains are an untapped and attractive source of novel secondary metabolites.

## Abbreviations

NBRC: Biological Resource Center, National Institute of Technology and Evaluation
NRPS: non-ribosomal peptide synthetase
PKS: polyketide synthase
C: condensation
A: adenylation
T: thiolation
KS: ketosynthase
AT: acyltransferase
ACP: acyl carrier protein
CoA: coenzyme A
Ser: serine
E: epimeration
Orn: ornithine
MT: methylation
Gly: glycine
Asp: asparagine acid
Thr: threonine
DH: dehydratase
KR: ketoreductase
ER: enoylreductase
Asn: asparagine
Leu: leucine
Val: valine
Phe: phenylalanine
Tyr: tyrosine
